# Innate Immunity Evasion by Dengue Virus

**DOI:** 10.3390/v4030397

**Published:** 2012-03-15

**Authors:** Juliet Morrison, Sebastian Aguirre, Ana Fernandez-Sesma

**Affiliations:** Department of Microbiology and the Global Health and Emerging Pathogens Institute (GHEPI), Mount Sinai School of Medicine, New York, NY 10029-6574, USA; Email: juliet.morrison@mssm.edu (J.M.); sebastian.aguirre@mssm.edu (S.A.)

**Keywords:** interferon antagonism, NS2B3, NS5, STAT2 degradation

## Abstract

For viruses to productively infect their hosts, they must evade or inhibit important elements of the innate immune system, namely the type I interferon (IFN) response, which negatively influences the subsequent development of antigen-specific adaptive immunity against those viruses. Dengue virus (DENV) can inhibit both type I IFN production and signaling in susceptible human cells, including dendritic cells (DCs). The NS2B3 protease complex of DENV functions as an antagonist of type I IFN production, and its proteolytic activity is necessary for this function. DENV also encodes proteins that antagonize type I IFN signaling, including NS2A, NS4A, NS4B and NS5 by targeting different components of this signaling pathway, such as STATs. Importantly, the ability of the NS5 protein to bind and degrade STAT2 contributes to the limited host tropism of DENV to humans and non-human primates. In this review, we will evaluate the contribution of innate immunity evasion by DENV to the pathogenesis and host tropism of this virus.

## 1. Introduction

Innate immunity and type I IFN responses function as the first line of defense against viral infections. Most viruses target these important elements to avoid being sensed or recognized in infected cells and to efficiently establish infection in the host. Several viruses have been shown to inhibit type I IFN production in infected cells, but it has been shown only recently by our group that DENV can target the IFN production pathway in infected cells as an additional immune evasion strategy. On the other hand, several DENV proteins have been identified in recent years to be involved in the inhibition of type I IFN signaling in infected cells. By inhibiting this important pathway, the virus is able to stop the induction of hundreds of IFN inducible genes with antiviral functions that may impair several aspects of the virus cycle.

DENV is a human pathogen that productively infects a wide range of cells involved in the immune response. It has been demonstrated that DENV replicates in monocytes, macrophages, B cells, and dendritic cells (DCs) among others. DCs are professional antigen-presenting cells (APCs) and also some of the first cells that interact with DENV upon the bite of an infected mosquito [30,35,36,71]. This encounter favors the internalization of DENV by receptor-mediated endocytosis after the adhesion of the structural E protein with cellular receptors such as dendritic cell-specific intracellular adhesion molecule-3-grabbing non-integrin (DC-SIGN). Acidification of endosomes induces an irreversible conformational change of the E protein, resulting in the fusion of endosomal and viral membranes followed by the release of the nucleocapsid into the cytoplasm. The DENV genome is composed of a single-stranded positive-sense RNA molecule that is translated into a single polyprotein of approximately 3391 amino acids. After translation, the polyprotein localizes within the ER membrane where it is cleaved by the viral protease complex, NS2B3, and cellular proteases to generate three structural proteins [capsid (C), premembrane/membrane (prM/M) and envelop (E)] and seven non-structural proteins (NS1, NS2A, NS2B, NS3, NS4A, NS4B and NS5). Some non-structural proteins have the ability to modify the ER membrane, creating the micro-environment necessary for the synthesis of new viral products. The assembly of these viral products invokes the formation of virions, which are subsequently exocytosed via Golgi-derived secretory vesicles.

In the present review, we discuss the recent reports that have shown and defined the possible mechanisms of inhibition of both the production and signaling of type I IFN by DENV. We also discuss the implications of DENV’s evasion of this important innate immunity mechanism on the development of antiviral drugs or animal models for this important human pathogen.

## 2. Discussion

### 2.1. Inhibition of Type I IFN Production by DENV

The accumulation of pathogen-associated molecular patterns (PAMPs) like the viral intermediate double-stranded RNA (dsRNA), facilitates virus detection by a series of host pathogen recognition receptors (PRRs) leading to the activation of professional APCs. The most relevant PRRs for the detection of DENV products, described so far are the membrane-bound Toll-like receptors (TLR3/TLR7/TLR8) and the cytosolic receptors (RIG-I/MDA-5) [43,50,65]. As with other intracellular pathogens, these PAMP/PRR interactions trigger a cascade of events that ends with the expression of a series of cytokines and chemokines of paramount importance for the control of the infection. Several groups have demonstrated that DENV is a weaker inducer of type I IFN responses after infection of human dendritic cells, with a minimal production of IFNα/β [27,57,61] especially compared to other viral infections that competently induce significant levels of these fundamental cytokines, as is the case for Newcastle disease virus (NDV) [20] and Semliki Forest virus (SFV) [25]. The absence of type I IFN production by DENV-infected DCs results in an impaired ability of those DCs to prime T cells toward Th1 immunity, an effect that can be reversed by the addition of exogenous IFNβ [57]. Interestingly, DENV is able to trigger the expression of some proinflammatory cytokines (IL-6, IL-8, TNFα, and MIP-1β), a phenomenon observed at early times after infection. An explanation for this hallmark feature of DENV is that the virus acquired, through evolution, a strategy to attract target cells to the site of infection by allowing the expression of some chemoattractant molecules by infected cells. 

**Figure 1:**
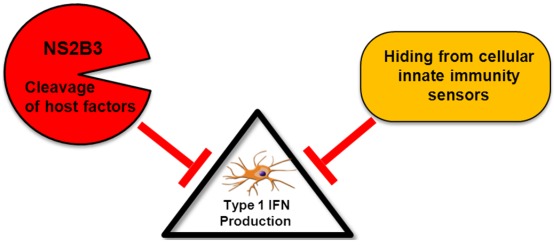
Model for inhibition of type I interferon (IFN) production by DENV: Dengue virus (DENV) can inhibit the induction of type I IFN using different strategies. The NS2B3 protease complex targets and cleaves still unidentified components of the type I IFN production pathway leading to the interruption of IRF3 phosphorylation. Furthermore, DENV can evade encounter with cellular sensors by hiding its replication products inER-derived membranes.

DENV has successfully evolved to overcome host innate immunity and productively infect the host using a balanced combination of two fundamental strategies (Figure 1), one is passively, by evading the interaction of PAMPs with the cellular PRRs, and the other is actively, by the inhibition of different steps of the innate immune response through the expression of antagonist molecules which directly block the intracellular pathways that lead to type I IFN production and signaling. 

#### 2.1.1. Passive Evasion of PRR Detection

DENV infection provokes virus-induced intracellular membrane structures including vesicle packets (VPs) and convoluted membranes (CMs) [13]. This intracellular arrangement provides a physical framework that concentrates the host and viral components required for virus replication. VPs are ER-bound vesicles of about 3.5 × 10^−4^ μm^3^ that contain multiple non-structural proteins, double-stranded RNA and *de novo* synthesized RNA and are connected by pores to the cytoplasm [21,44,69]. This spatial rearrangement creates a microenvironment that is partially isolated from the cytosol and some of its components. This feature, which is common to many other positive-sense RNA viruses, not only promotes the concentration of viral products necessary for replication, but also precludes the physical interaction with the host PRRs. This phenomenon could delay the PRR-PAMP interaction until the replication process synthesizes enough viral products for the constitution of the new progeny, a strategy also described for other flaviviruses [21]. In parallel, another important element in the evasion strategy is the conserved viral RNA conformation. DENV RNA secondary structure has an impact on replication in both mosquito and mammalian hosts [2]. Cis-acting elements required for RNA synthesis and translation initiation can be found at the 5′UTR and the 3′UTR of the viral genome [67]. Also, the RNA secondary structure at the flavivirus 3′UTR provides resistance to cellular RNases and promotes the accumulation of sub-genomic RNA with implications for cytopathicity in cell culture as well as pathogenicity in mice [22]. These specific conformations acquired through viral evolution could also be implicated in the poor detection of DENV by the PRRs. The rational modification of these structures at the untranslated regions by point mutations or deletions would be an interesting approach to evaluate the viral RNA/PRR interaction.

#### 2.1.2. Active Inhibition of Type I IFN Production by DENV

**Figure 2:**
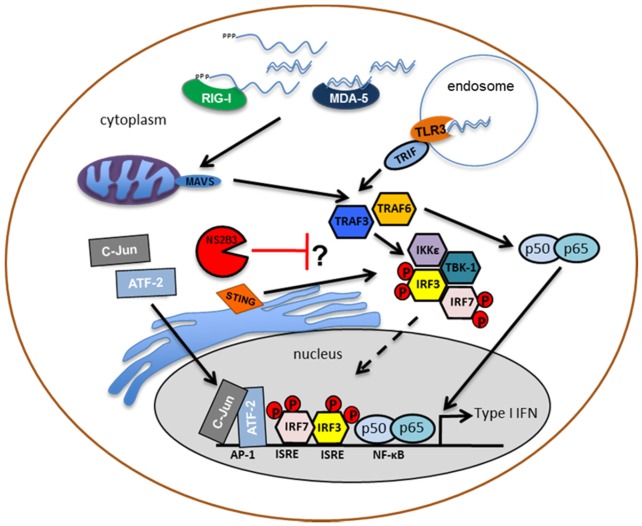
Inhibition of type I IFN production by DENV: The recognition of DENV replication intermediates by the cytosolic helicases, RIG-I and MDA-5, induces a conformational change that promotes the interaction between the caspase-recruitment domain (CARD) of these pattern-recognition receptors and the CARD-containing adaptor, MAVS. Furthermore, the membrane-bound protein, Toll-like receptor 3 (TLR3), binds dsRNA in endosomes, inducing the activation of the adaptor protein, TRIF. All of these pathways converge in the recruitment and activation of the IKKε/TBK-1 complex. This activation induces phosphorylation and homo- and hetero-dimerization of transcription factors, IRF3 and IRF7, which translocate to the nucleus to induce the expression of type I interferon together with activated NF-κB and AP-1. The DENV protease complex inhibits the phosphorylation of IRF3 thereby resulting in a lack of type I IFN production in human dendritic cells.

Frequently, viruses are able to express proteins that interfere with the type I IFN response. A few of them in particular, can actively inhibit the production of IFN-α/β using this strategy. Some of the most studied are the influenza A NS1 protein [23] and the VP35 protein of Ebola virus [6]. In the case of DENV, our group demonstrated, using a primary human cell system, that infection of human DCs with DENV did not induce IRF-3 phosphorylation, resulting in an inhibition of type I IFN production after DENV infection [58] (Figure 2). A subsequent report, also by our group, explored the ability of DENV-infected DCs to respond to a variety of type I IFN-triggering signals using potent stimulators such as NDV, SeV, SFV, or TLR3 ligand poly(I:C) [57] (illustrated in Figure 2). These two studies, demonstrated that DENV-infected DCs failed to produce type I IFN and that these have reduced type I IFN production upon secondary infection or stimulation. This effect is viral dose dependent and takes place as early as 2 hours after DENV infection. We also showed that the inhibition of IFNα/β production after NDV infection in DENV-infected DCs is not a bystander effect, implying an active role of the DENV-infected DC population in the inhibition of IFNα/β. By using an NDV vector strategy to express the DENV non-structural proteins (NS2A, NS2B3, NS4A and NS4B), it was shown that the only recombinant NDV that triggered diminished IFNα expression was the one carrying the NS2B3 protease complex. Similar results were seen using an IFNβ promoter activity assay [57]. Catalytically inactive NS2B3 mutants showed a diminished inhibition of this phenotype, suggesting an important role for the protease activity of the NS2B3 protein as an innate immunity antagonist [57]. Interestingly, the proteolytic core of NS2B3, consisting of the last 40 amino acids of NS2B and the first 180 amino acids of NS3, was enough to reduce the activity of the IFNβ promoter [57]. Further studies are needed to decipher the exact mechanism of inhibition of type I IFN production by DENV in infected cells and to identify the host partners that are involved in this inhibition. Identification of host factors that contribute to this inhibition by interacting with or by being cleaved by DENV NS2B3 protease will be crucial for the design of antiviral drugs and intervention steps for DENV infection.

### 2.2. Inhibition of Type I IFN Signaling by DENV

**Figure 3:**
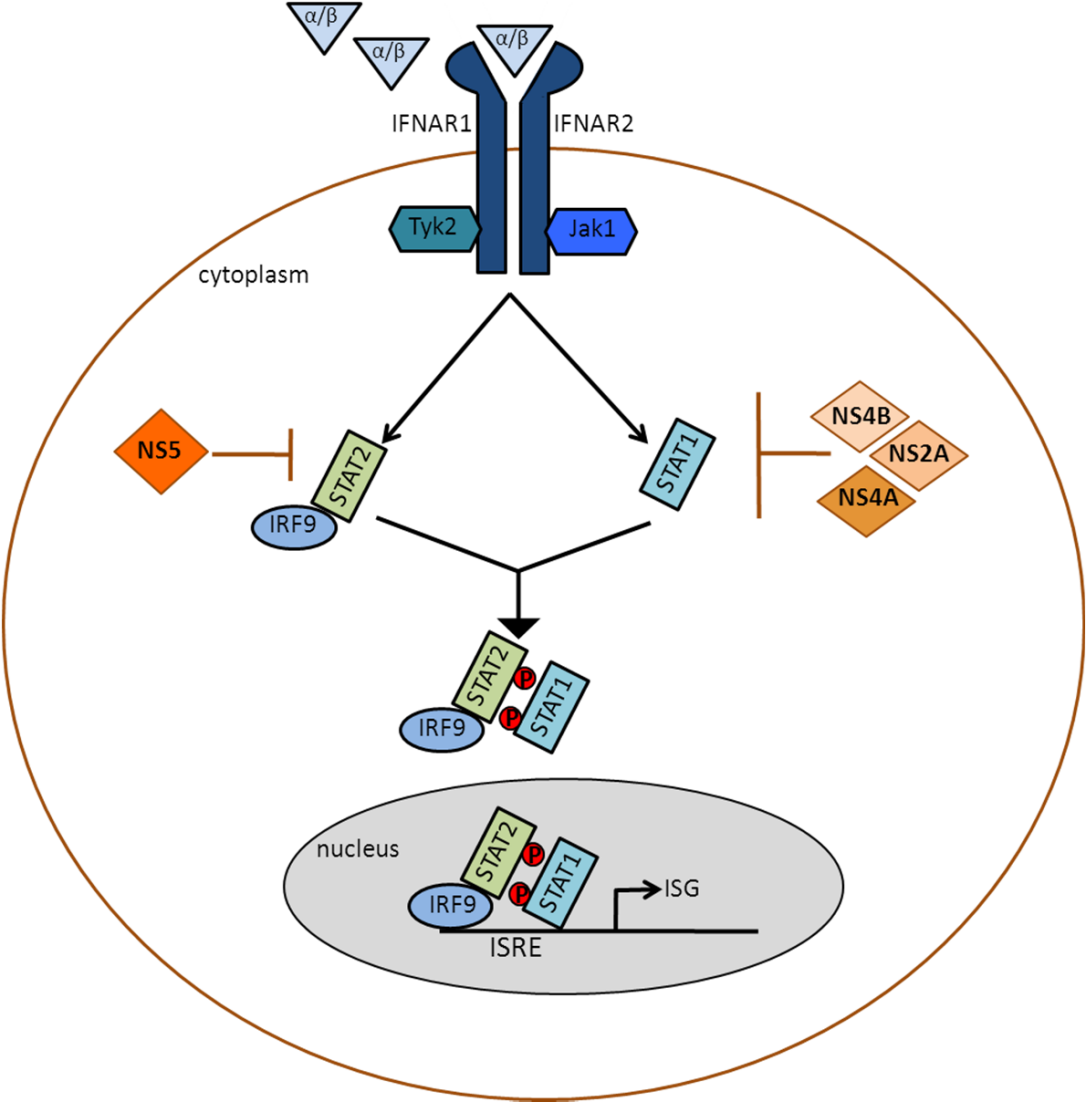
Inhibition of type I IFN signaling by DENV: IFNα/β binds the type I IFN receptor (IFNAR), which is composed of IFNAR1 and IFNAR2 and is found on the surface of nucleated cells. Receptor binding triggers tyrosine autophosphorylation of the Janus kinases, Jak1 and Tyk2. The activated kinases then phosphorylate STAT1 and STAT2. Activated STAT2, which is normally associated with IFN regulatory factor 9 (IRF9), associates with activated STAT1 to form the trimeric IFN-stimulated gene factor 3 (ISGF3) complex, which translocates to the nucleus and binds to the IFN-stimulated response element (ISRE) of IFN-stimulated genes (ISGs). Hundreds of ISGs are transcribed, and their products set up an antiviral state in the IFN-treated cell. DENV antagonizes this signaling pathway by utilizing NS5 to degrade STAT2, and by using NS2A, NS4A and NS4B to inhibit steps upstream of STAT1 activation.

Type I IFN signaling is initiated when IFNα/β binds to type I IFN receptors (IFNAR) that are present on the surface of most cells. This results in the activation of the receptor-associated tyrosine kinases, Janus kinase 1 (Jak1) and tyrosine kinase 2 (Tyk2), which subsequently phosphorylate signal transducer and activator of transcription 1 (STAT1) and signal transducer and activator of transcription 2 (STAT2). Phosphorylated STAT1 and STAT2 along with interferon regulatory factor 9 (IRF9) form a heterotrimeric complex known as IFN-stimulated gene factor 3 (ISGF3) that translocates to the nucleus and binds the IFN-stimulated response element (ISRE) found upstream of IFN-stimulated genes (ISGs) [11] (Figure 3). Hundreds of ISGs exist, and many of their products have been shown to possess antiviral activities. These include protein kinase R(PKR), viperin, the 2′,5′-oligoadenylate synthetase (OAS) family and the IFN-induced transmembrane protein (IFITM) family. The importance of type I IFN signaling in controlling DENV replication and disease is emphasized by experiments conducted in IFNAR knock out (KO) mice and STAT2 KO mice [5,32,55,60]. Though wild-type mice are unable to support DENV replication, DENV can replicate in IFNAR KO and STAT2 KO mice. High viral loads and dengue disease symptoms are only seen in mice that lack both the type I and type II IFN pathways, indicating that the type II IFN response is also important in limiting viral replication [9,32,55,60]. DENV inhibits IFNα/β signaling, and its NS2A, NS4A, NS4B and NS5 proteins have been implicated in this process [4,45,48,49].

Type II IFN signaling is distinct from type I IFN signaling. Type II IFN or IFN gamma (IFNγ) is produced only by immune cells, and binds the IFNγ receptor (IFNGR), which is located on a variety of cell types. Binding of IFNγ to its receptor leads to the activation of receptor-associated Jak1 and Jak2. This results in the phosphorylation of STAT1 and the formation of a STAT1 homodimer called IFNγ-activated factor (GAF), which translocates to the nucleus and binds to the IFNγ-activated sites (GAS) in a subset of genes [11]. This gene subset contains many of the genes that are activated by type I IFN signaling, but some genes are exclusively activated by only one of the IFN-signaling cascades [12,14]. Though there is strong evidence for DENV-mediated inhibition of IFNα/β signaling, IFNγ-signaling does not appear to be targeted by the virus [26,33,66]. This contrasts with related viruses such as West Nile virus (WNV) and Langat virus (LGTV) that inhibit both pathways [7,42]. 

Many of the type I IFN signaling components are also shared by the type III IFN response. In this signaling cascade, type III IFN or IFN lambda (IFNλ) binds to its receptor (IL10Rβ and IL28Rα), which though structurally different from IFNAR, is also associated with Jak1 and Tyk2 and results in the formation of ISGF3 when activated [3]. Unlike IFNAR, IL28R is found only on a subset of cells [74]. The contribution of type III IFN to DENV host responses has not been studied though type III IFN has recently emerged as a determinant of patient outcomes during infection with another *Flaviviridae* family member, hepatitis C virus [24,62,64]. It is currently unknown if DENV antagonizes type III IFN signaling.

#### 2.2.1. Pretreatment with Type I IFN Can Inhibit DENV

Though DENV can replicate in cells if IFN treatment occurs post infection, IFNα/β inhibits DENV replication if it is added prior to infection [15,16]. This inhibition is independent of PKR and RNaseL since PKR and RNaseL double knockout mouse embryonic fibroblasts (MEFs) inhibited DENV replication as well as wild-type MEFs [15]. A variety of ISGs have been implicated in DENV suppression. Overexpression of viperin, an ISG that is highly expressed during DENV infection, inhibits DENV replication in A549 cells. Tumor necrosis factor-related apoptosis inducing ligand (TRAIL), which is induced by type I IFN signaling and upregulated during DENV infection, inhibits DENV replication in human dendritic cells [68]. IFITM1, IFITM2 and IFITM3 restrict early steps in both DENV and WNV replication [8]. Jiang and colleagues recently confirmed PKR’s irrelevance in IFN-mediated inhibition of DENV replication, as well as the involvement of viperin, IFITM2 and IFITM3 in virus inhibition [31]. They also identified ISG20 as an inhibitor of DENV replication [31]. Many previously uncharacterized ISGs have now been shown to inhibit flaviviruses such as yellow fever virus (YFV) and WNV [59]. It is likely that other ISGs that inhibit DENV will be identified as more of them are screened.

#### 2.2.2. DENV Inhibits Type I IFN Signaling

When various cell lines were pretreated with IFNα/β, IFNγ or a combination of both, replication of several low- and high-passage DENV 2 strains was inhibited [16]. Interestingly, the effect of IFNα and IFNβ were far more inhibitory than the effects of IFNγ, and the virus appeared to escape most of this inhibition if cells were infected prior to IFNα/β treatment. Several reports have shown that human and non-human primate cells infected with DENV or harboring dengue replicons have decreased type I IFN signaling [4,26,33,45,49].

Infection with DENV decreases type I IFN-mediated STAT1 phosphorylation and also causes a proteasome-dependent decrease in STAT2 protein levels [4,26,33,49]. Type I IFN-induced STAT1 phosphorylation was reduced in DENV2-infected A549 cells [49] and DENV2-infected K562 cells [33]. K562 cells containing a DENV2 replicon have decreased STAT1 phosphorylation as well as decreased STAT2 levels [33,45]. Vero cells containing a DENV1 replicon or infected with DENV2 also showed a decrease in STAT2 levels, and this decrease was proteasome-dependent [4]. A decrease in Tyk2 and STAT1 phosphorylation has also been documented in DENV2-infected dendritic cells though no STAT2 degradation was observed in that study [26]. The variation observed with regards to STAT1 phosphorylation, Tyk2 phosphorylation and STAT2 degradation may be due to the levels of viral replication in different cell types as well as virus strain and cell-type dependent differences. In fact, A549 and HepG2 cells showed differential type I IFN responses to two DENV2 strains, NGC and TSVO1, with NGC but not TSV01, inhibiting STAT1 phosphorylation [66]. Furthermore, though NGC-induced STAT2 degradation was not observed in A549 and HepG2 cells in that paper, Jones and colleagues showed that NGC induced STAT2 degradation in K562, supporting the idea of cell-type specific differences [33]. Though strain and cell-type dependent differences do appear to exist, there are three common observations seen when DENV infects human and non-human primate cells: type I IFN signaling is impaired, type I IFN-mediated STAT1 phosphorylation is inhibited, and total STAT2 protein levels decrease. We will now explore the contribution of different viral proteins to these processes.

DENV encodes at least four proteins that inhibit type I IFN signaling (illustrated in Figure 3). Consequently, it can replicate in type I IFN-treated cells when cells are treated post infection [16]. This section will discuss how DENV inhibits type I IFN signaling through the use of the nonstructural proteins NS2A, NS4A, NS4B and NS5. We will also describe the contribution of NS5-mediated IFN antagonism to the limited host tropism of the virus, and how our understanding of this phenomenon may eventually lead to the development of an immune-competent mouse model of dengue infection.

##### 2.2.2.1. NS2A-, NS4A- and NS4B-Mediated Inhibition of STAT1 Phosphorylation

Flavivirus NS2A, NS4A and NS4B are small hydrophobic proteins that associate with cellular membranes. DENV NS2A is thought to be involved in virus assembly because of its sequence similarity to NS2A proteins of Kunjin (KUN) virus and yellow fever virus (YFV), which are required for virion assembly [38,40]. DENV NS4A induces the membranous structures that house the viral replication complexes and colocalizes with dsRNA and structural protein E [46]. DENV NS4B also colocalizes with E and dsRNA suggesting that it is a part of the replication complex [47]. In addition to their roles in viral replication, NS2A, NS4A and NS4B of DENV have also been implicated in IFN signaling inhibition [48,49].

DENV-infected A549 cells have decreased STAT1 phosphorylation and IFN-induced ISRE-promoter activation [49]. This effect could be recapitulated when NS2A, NS4A and NS4B were expressed individually in cells, with NS4B inhibiting the pathway most potently. A more potent antagonistic effect was seen when the proteins were expressed together. NS4B’s ability to inhibit IFN signaling depends on its proper targeting to the ER membrane by the 2K protein [48]. NS4B proteins from other flaviviruses also inhibit type I IFN signaling [19,48]. Interestingly, a WNV NS4B mutation that decreased inhibition of type I IFN-signaling when overexpressed from a plasmid or in the context of a replicon had no IFN signaling antagonist defect in the context of the full virus [19]. Thus, with regards to IFN signaling inhibition, interplay between viral proteins exists during WNV infection, and this may also be the case for DENV.

##### 2.2.2.2. NS5-Mediated Degradation of STAT2

Flavivirus NS5 is a 105-kDa protein that encodes an RNA guanylyltransferase, a methyltransferase (MTase) and an RNA-dependent RNA polymerase (RdRp). The N-terminus encodes an RNA guanylyltransferase that, in conjunction with NS3, initiates 5′ cap formation starting with a GTP substrate [29]. The N-terminus also encodes a methyltransferase that methylates guanine N-7 and ribose 2′-O of the viral RNA cap [17,18]. In both DENV and WNV, N-7 methylation has been shown to be more critical for viral replication than 2′-O methylation [17,73]. WNV mutants that lack 2′-O methylation function are sensitive to the antiviral effects of the IFN-induced tetratricopeptide repeat (IFIT) family members [10]. These data indicate that WNV NS5 inhibits the antiviral actions of a subset of ISGs, and can also be considered an ISG antagonist. This may also be the case for DENV NS5 since 2′-O methylation mutants similar to those in WNV also show replication defects [17]. The carboxy terminal region of NS5 interacts with the NS3 protein [34] and encodes the RdRp domain of DENV [1,51,63]. The multifunctional role of NS5 is demonstrated with mutants that lack one function but retain others. For example, an NS5 mutant can lack NS3 binding capabilities while retaining enzymatic activity, and other mutations inhibit viral replication while maintaining polymerase function [28,75]. Differential phosphorylation of NS5 may serve as a switch between these functions [34].

**Figure 4:**
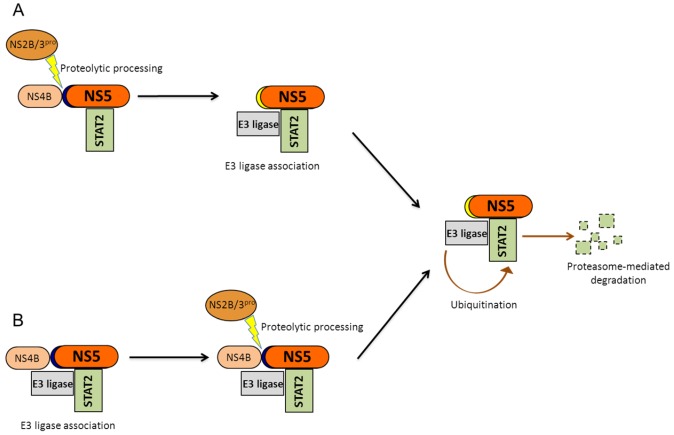
Potential mechanisms for NS5-mediated STAT2 degradation: STAT2 degradation requires that NS5 be cleaved at its amino terminus. (**A**) During infection, cleavage of NS5 may activate NS5 to direct STAT2 to an E3 ligase which would then target STAT2 to the proteasome for degradation;(**B**) Alternatively, the NS5 precursor may associate with an E3 ligase and STAT2, and subsequent cleavage of NS5 away from NS4B would trigger E3 ligase activation and STAT2 degradation.

The NS5 proteins of several flaviviruses have been shown to inhibit IFN signaling. These include Langat virus, tick-borne encephalitis virus, Japanese encephalitis virus, DENV and West Nile virus [4,7,39,41,45,54,70]. DENV NS5 antagonizes IFN signaling by binding and degrading STAT2. When NS5 is expressed during virus infection, during replicon replication, or from a plasmid encoding NS1 through 5, cellular STAT2 levels diminish [4,33,45]. This observation was attributed to NS5 since constructs expressing all of the nonstructural proteins but NS5 were unable to decrease STAT2 levels [4]. NS5 binds STAT2 and inhibits type I IFN signaling when it is expressed alone but is unable to mediate STAT2 degradation [4,45] (Figure 4). Ashour and colleagues ascribed this to a difference between NS5 that is proteolytically cleaved from a precursor protein *versus* NS5 that is produced alone from a plasmid [4]. During a viral infection, NS5 is cleaved away from NS4B by NS2B3 protease. The researchers observed STAT2 degradation in cells expressing NS5 downstream of a NS2B3 protease cleavage recognition site in conjunction with NS2B3 protease, or NS5 downstream of a tobacco etch virus (TEV) protease recognition cleavage site in conjunction with TEV protease. STAT2 degradation also occurred in cells expressing NS5 downstream of ubiquitin. Because ubiquitin is cleaved away from downstream sequences by cellular hydrolases, a ubiquitin-NS5 construct produces a cleaved NS5 protein that resembles that seen during DENV infection. Thus STAT2 degradation requires that NS5 is cleaved at the amino terminus but is independent of the protease used to cleave NS5 [4]. During infection, cleaved NS5 may direct STAT2 to an E3 ligase that targets it to the proteasome for degradation. Alternatively, the NS5 precursor may associate with an E3 ligase and STAT2, and subsequent cleavage of NS5 away from NS4B could activate STAT2 degradation (Figure 4).

#### 2.2.3. Type I IFN Contributes to the Limited Host Tropism of DENV

Though DENV replicates in humans and causes hemorrhagic disease, it is unable to do so in mice unless they lack components of the IFN response. DENV is able to replicate in mice that lack receptors for type I IFN signaling, or the type I signaling component STAT2 [5,32,55,60]. However, optimal replication and hemorrhagic symptoms are only seen when both the type I and type II IFN responses are dismantled [55,60]. The ability of DENV to inhibit human but not murine type I IFN can be explained by the affinity of NS5 for human STAT2 (hSTAT2) but not murine STAT2 (mSTAT2) [5]. This is reminiscent of parainfluenza virus 5 (formerly known as simian virus 5) that antagonizes type I IFN signaling in human cells but not in murine cells, because the viral V protein can associate with hSTAT2 but not mSTAT2 to target STAT1 for degradation [52]. When STAT2-deficient human cells are reconstituted with mSTAT2, they are inhibitory to DENV replication in an IFNα/β-dependent manner. However, when the cells are reconstituted with hSTAT2, DENV replication is not inhibited. Furthermore, when STAT2-deficient MEFs were reconstituted with hSTAT2, dengue replication was unaffected by IFNα/β treatment, but it was inhibited in MEFs expressing mSTAT2 [5]. This suggests that it may be possible for DENV to replicate in mice that express hSTAT2 in place of mSTAT2, especially since STAT2-deficient mice allow replication of DENV [5,55]. 

Because hSTAT2 allows parainfluenza virus 5 V protein to target STAT1 and inhibit type I IFN signaling, mice that ectopically express hSTAT2 support replication of parainfluenza virus 5 [37]. Since mSTAT2 is inhibitory to DENV replication, such a mouse would not support DENV replication. A mouse with hSTAT2 and no mSTAT2 may however support DENV replication, and would be immune-competent, since mSTAT2 and hSTAT2 are interchangeable for IFN signaling and ISG production [53]. The most widely used model of dengue infection, the AG129 mouse, lacks both type I and type II IFN signaling, so the contribution of the innate immune system to the development and resolution of dengue infections cannot be readily studied [56,72]. Humanized mice that are engrafted with CD34+ human cells form a human-like immune system, and do support the replication of DENV, but engraftment is highly variable [72]. A clonal population of immune-competent mice that are susceptible to DENV infection would be a valuable tool for investigating dengue infections, allowing researchers to study both immunology and immunopathology. A knock-in mouse expressing human STAT2 in place of murine STAT2 would likely be immune-competent and would likely allow replication of DENV as is seen in STAT2 KO mice. This animal could serve as a platform for investigation of the other causes of viral restriction namely the type II IFN response, eventually leading to the development of an immune-competent mouse model of dengue disease.

## 3. General Conclusions

The combination of effective inhibition of type I IFN production and signaling in infected cells and effective modulation of DC activation by DENV may contribute to the induction of suboptimal adaptive immunity in patients. Additionally, DENV efficiently evades immune recognition by limiting the exposure of genetic RNA, which would be strongly detected by viral sensors in infected cells. Another possible hypothesis is that DENV has evolved to contain specific RNA structures that are poorly detected by those sensors. In conclusion, DENV is very efficient at modulating innate immunity and this property allows the virus to successfully establish infection in humans. Understanding the early events that occur when DENV interacts with and infects antigen-presenting cells such as DCs will provide crucial information for the design of strong antivirals and vaccines against this important pathogen. Also, the identification of host factors targeted by DENV for the inhibition of type I IFN responses will prove very useful for the development of immune-competent animal models for dengue disease.
